# Molecular Characterization of the *ORF3* and *S1* Genes of Porcine Epidemic Diarrhea Virus Non S-INDEL Strains in Seven Regions of China, 2015

**DOI:** 10.1371/journal.pone.0160561

**Published:** 2016-08-05

**Authors:** Enyu Wang, Donghua Guo, Chunqiu Li, Shan Wei, Zhihui Wang, Qiujin Liu, Bei Zhang, Fanzhi Kong, Li Feng, Dongbo Sun

**Affiliations:** 1 College of Animal Science and Veterinary Medicine, Heilongjiang Bayi Agricultural University, Sartu District, Daqing, P.R. China; 2 Division of Swine Infectious Diseases, National Key Laboratory of Veterinary Biotechnology, Harbin Veterinary Research Institute of the Chinese Academy of Agricultural Sciences, Nangang District, Harbin, P.R. China; Fudan University, CHINA

## Abstract

In an effort to trace the evolution of porcine epidemic diarrhea virus (PEDV), *S1* and *ORF3* genes of viruses identified in 41 pig farms from seven regions (North, Northeast, Northwest, Central, East, South West, and South, respectively) of China in 2015 were sequenced and analyzed. Sequence analysis revealed that the 41 *ORF3* genes and 29 *S1* genes identified in our study exhibited nucleotide homologies of 98.2%–100% and 96.6%–100%, respectively; these two genes exhibited low nucleotide sequence similarities with classical CV777 strain and early Chinese strain LZC. Phylogenetic analysis indicated that the identified PEDV strains belonged to global non S-INDEL strains, and exhibited genetic diversity; *S1* gene of the HLJ2015/DP1-1 strain harbored an unique deletion of 12 nucleotides (A^1130^CAACTCCACTG^1141^); while the Chinese PEDV S-INDEL reference strains included two types of the “CV777” S-INDEL as well as the “US” S-INDEL, and all co-circulated with Chinese non S-INDEL strains. Of 29 identified *S1* genes, the SS2 epitope (Y^748^SNIGVCK^755^) was highly conserved, while the SS6 epitope (L^764^QDGQVKI^771^) and pAPN receptor-binding region (aa 490–615) exhibited amino substitutions. Nine possible recombination events were identified between the 29 identifed *S1* genes and the 3 *S1* reference genes from early Chinese PEDV strains. The complete *S* genes of selected Chinese PEDV field strains (2011–2015) showed 5.18%–6.07% nucleotide divergence, which is far higher than the divergence observed in early Chinese PEDV strains (3.1%) (*P*<0.05). Our data provide evidence that PEDV non S-INDEL strains with genetic diversities and potential recombination circulate in seven regions of China in 2015; Chinese PEDV S-INDEL strains exhibit genetic diversity and co-circulate with non S-INDEL strains.

## Introduction

Porcine epidemic diarrhea virus (PEDV), belonging to the order *Nidovirales*, the subfamily *Coronaviridae*, and the genus *Alphacronavirus*, is an enveloped, single-stranded, positive-sense RNA virus. PEDV was identified as the causative agent of porcine epidemic diarrhea (PED) in 1978 [1]. This disease is characterized by severe diarrhea, vomiting and dehydration. PEDV infections can occur in pigs of any age, but infections are most serious in piglets, with morbidity and mortality often reaching 100% [2]. At the end of 2010, a PEDV outbreak occurred in several pig-producing provinces in southern China. Since then, the disease has spread throughout other provinces of China and has resulted in enormous economic losses within the pork industry [3]. Thus far, PEDV infection has been reported in swine-farming countries in Asia, Europe, and North America [4–11].

The highly pathogenic PEDV variant was reported in China in late 2010, and since then there has been wide-spread epidemiological investigation of PEDV variant stains [4]. In December 2013, an S-INDEL strain, OH851, with reduced virulence was reported in the USA. Strain OH851 showed the same insertions and deletions in the spike gene (called S-INDEL) as the classical CV777 strain when compared with highly virulent US prototype strains [12, 13]. Yamamoto *et al*., (2015) reported one PEDV S-INDEL strain with a low pathogenicity in Japan [14]. While Stadler *et al*., (2015) reported that the S-INDEL strains, similar to the OH851 strain, were identified in southern Germany [15]. Boniotti *et al*., (2016) reported a new swine enteric coronavirus generated by recombination of PEDV and swine transmissible gastroenteritis virus (TGEV) [9]. At present, both S-INDEL and non S-INDEL strains co-circulate in the USA, China, and Japan [14, 16]. The accumulating data have revealed the genetic diversity and complex change tendencies of PEDV strains circulating in a global context.

In the majority of PEDV strains, *ORF3* gene is highly conserved, while the *S1* region of the *S* gene (*S1*) shows high variability [17, 18]. As a result of the high sequence variation in the *S1* gene, the more conserved *ORF3* gene is widely used for diagnosis of PEDV infection. While the more variable *S1* gene has been widely applied to studies of PEDV genetic diversity and evolution. Although researchers have continued to do epidemiological monitoring of PEDV infections in China, there is fairly limited information available on currently circulating PEDV viruses in 2015. In this study, molecular epidemiological investigation of PEDV was carried out in seven regions of China in 2015. We used the *ORF3* and *S1* genes to analyze the genetic evolution of the identified PEDV strains. Our aim was to provide insights into the genetic evolution of PEDV strains circulating in China in 2015.

## Materials and Methods

### Ethics Statement

The collection of samples from the diseased pigs used in this study was approved by the Animal Care and Use Committee of the Harbin Veterinary Research Institute at the Chinese Academy of Agricultural Sciences in China. The field study did not involve endangered or protected species. No specific permissions were required for locations of samples because the samples were collected from public areas or non-protection areas. The sampling and data publication also were approved by owners of the pig farms.

### Collection of PEDV-positive samples

In our study, a total of 165 intestinal tissues samples were obtained from the diseased piglets of 41 PEDV-positive farms in 18 provinces or municipalities of China in 2015. The intestinal tissues samples of the dead animals were collected by veterinarians of each farm, and were stored at −20°C. The diseased piglets exhibited the clinical manifestations of acute diarrhea and vomiting. The antibiotic therapy was not effective for the diseased animals. The 41 farms covered seven administrative districts of China including the Northeastern, Northern, Northwestern, Southwestern, Central, Eastern and Southern regions. Farms were selected as PEDV-positive using the colloidal gold immunochromatographic rapid test strip for PEDV (BioNote, Inc., Republic of Korea) or using RT-PCR. The PEDV infection status of all 165 intestinal samples was further confirmed using the conventional RT-PCR method which targets the *ORF3* gene [19]. Briefly, 1 g of intestinal tissue was placed in 5 ml of PBS (pH 7.4), ground up and clarified. The viral RNA was then extracted from the supernatant of each sample using the TIANamp Virus RNA Kit (Tiangen Biotech Co., Ltd., Beijing, China) according to the manufacturer’s instructions. The viral cDNA was synthesized by using Moloney murine leukemia virus (RNase H-) reverse transcriptase (Novoprotein Scientific Inc., Shanghai, China) in conjunction with six random nucleotide primers according to manufacturer’s instructions. The PCR amplification of the *ORF3* gene was carried out according to the protocol described by Kim *et al*., (2015) [19]. One positive sample from each farm was selected for sequencing of the *ORF3* gene. All nucleotide sequences generated in our study were submitted to GenBank. Sequence analysis was performed using the EditSeq tool in Lasergene DNASTAR^™^ 5.06 software (DNASTAR Inc., Madison, WI, USA). Multiple sequence alignments were performed using the Multiple Sequence Alignment tool of DNAMAN 6.0 software (Lynnon BioSoft, Point-Claire, Quebec, Canada).

### Sequencing and analysis of the PEDV S1 gene

To obtain the *S1* gene of PEDV, a pair of specific primers, PEDVS1-F: 5' TTTTCTAATCATTTGGTCAACG 3' and PEDVS1-R: 5' AATACTCATACTAAAGTTGGTGG 3', were designed to amplify the sequence between nucleotide 20609 and 23004 of the PEDV genome. This region was chosen based on the alignment of published PEDV genome sequences obtained from the NCBI GenBank database. The alignment revealed a 2396 bp fragment (partial *nsp13* gene +*S1* gene) covering the *S1* gene. The primer positioning was determined according to the complete genome sequence of PEDV strain CV777 (GenBank accession no. AF353511). PCR amplification was undertaken in 50 μL reaction volumes containing 0.1 μM of forward primer, 0.1 μM of reverse primer, 4 μL of cDNA (synthesized as described previously), 5 μL of 10 × buffer, 0.5 U of ExTaq DNA polymerase (TaKaRa), and an appropriate volume of ddH_2_O. PCR products were amplified using the following cycle conditions: 30 cycles of 95°C for 1 min, 51°C for 1 min, and 72°C for 3 min, with a final extension at 72°C for 10 min. The reaction was carried out by using an automated GeneAmp PCR System 9700 thermal cycler (Applied Biosystems). The purified PCR products were subjected to Sanger sequencing, and all nucleotide sequences generated in our study were submitted to GenBank. A total of 29 *S1* genes, corresponding to the previously sequenced *ORF3* genes, were obtained. The epitopes of these 29 *S1* genes were analyzed using SS2 (Y^748^SNIGVCK^755^) SS6 (L^764^QDGQVKI^771^) as described by Sun *et al*., (2008) [20]. These 29 *S1* genes were compared with the PEDV S-INDEL reference strains, and assigned as S-INDEL or non-INDEL strains [16]. The amino acid substitutions in the pAPN receptor-binding region (aa 490–615) of PEDV S1 protein were analyzed and compared to the reference strains [17]. Finally, recombination analysis of the 29 identified *S1* genes was carried out using the RDP4 software with parameters similar to those described by Geng *et al*., (2015) [21, 22].

### Phylogenetic analysis

For the phylogenetic analysis, the *ORF3* and *S1* genes of PEDV strains from different geographical locations within China and the rest of the world were retrieved from the NCBI nucleotide database. Nucleotide sequences of the *ORF3* genes are shown in Supporting Information (S1 Fig); nucleotide sequences of the *S1* genes are shown in Supporting Information (S2 Fig). These nucleotide sequences were used to generate a neighbor-joining phylogenetic tree of the *ORF3* gene or *S1* gene using the ClustalX alignment tool in the MEGA6.06 software [23]. Neighbor-joining phylogenetic trees were built using the *p*-distance model and 1000 bootstrap replicates.

### Analysis of S gene variation in PEDV

To explore the genetic variation of PEDV strains, the nucleotide sequences of the entire *S* gene of selected Chinese PEDV strains were retrieved from the NCBI nucleotide database. The nucleotide divergence of all selected *S* genes to the *S* gene of the prototype CV777 strain (Genbank accession no. AF353511) were calculated by using the MegAlign tool included in the Lasergene DNASTAR^™^ software package (version 5.06). The selected *S* genes were divided into six groups based on the collection date. These analyses revealed the following: (1) four were early Chinese PEDV strains (Genbank accession no. AY653204/ EF185992/ EU031893/JN547228); (2) 22 were 2011 strains (Genbank accession no. JQ239429-JQ239436/JQ282909/JQ979287-JQ979290/JX188454/JX188455/JX524137/KC196276/KC242897/KP399631-KP399634); (3) 19 were 2012 strains (Genbank accession no. JX112709/JX560761/KC242898-KC242912/KF384500/KP399630); (4) 29 were 2013 strains (Genbank accession no. KF601195-KF601201/KF761675/KJ020932/KP399610-KP399629); (5) 16 were 2014 strains (Genbank accession no. KM242131/KP399601-KP399609/KP890336/KR296665/KR296672-KR296675); and (6) 19 were unique 2015 strains (Genbank accession no. KR095279/KR296663/KR296664/KR296666-KR296671/KR296676-KR809885/KT199103).

### Statistical analysis

All data sets were analyzed using a two-tailed, paired Student’s *t*-test in Microsoft Excel 2007 software. The nucleotide divergence (%) of each group is presented as “average value ± standard deviation”. The different letters (one or more) represent statistically significantly difference (*P*<0.05) among six groups of the early Chinese PEDV strains (n = 4), PEDV strains from 2011 (n = 22), PEDV strains from 2012 (n = 19), PEDV strains from 2013 (n = 29), PEDV strains from 2014 (n = 16), and PEDV strains from 2015 (n = 19); the same letters (one or more) represent no significant difference (*P*>0.05) among six groups.

## Results

### Sequencing and analysis of ORF3 and S1 genes of PEDV

In the current study, 165 samples from 41 PEDV-positive farms in 18 provinces or municipalities of China were identified by RT-PCR or rapid strip test. Of those 165 samples, 137 (83.03%) were confirmed as PEDV positive using by RT-PCR targeting the *ORF3* gene of PEDV (Table 1). A total of 41 independent *ORF3* genes and 29 *S1* genes were successfully sequenced, covering seven regions of China. The sequence comparisons of the *ORF3* genes revealed nucleotide homologies of 98.2%–100% and amino acid homologies of 97.8%–99.6% among the 41 PEDV strains; the 41 identifed PEDV strains exhibited 96.4%–97.2% nucleotide sequence homologies with the prototypical CV777 strain (Table 2). Sequence comparisons of the 29 identified *S1* genes indicated nucleotide homologies of 96.6%–100% and amino acid homologies of 95.2%–99.5%, while nucleotide and amino acid homologies of 92.0%–92.7% and 89.5%–91.3% respectively, were observed between the 29 identifed PEDV strains and the prototypical CV777 strain (Table 2). These data demonstrated that both the *ORF3* and *S1* gene of the identified PEDV strains exhibited high sequence similarity to Chinese highly virulent PEDV strain CHYJ130330, Chinese recombinant PEDV strain CH/HNQX-3/14, and US prototype strain USA/Colorado/2013. They also showed low sequence similarity to the CV777 strain (*ORF3*: 94.7%–96% amino acids identity; *S1*: 89.5%–91.3% amino acids identity), early Chinese PEDV field strain LZC (*ORF3*: 93.3%–94.2% amino acids identity; *S1*: 89.2%–90.5% amino acids identity) (Table 2).

**Table 1:** Information about PEDV-positive samples identified in our study.

Farm no.	Strain name	Collection date	Geographic location (City/Province/Region*)	GenBank accession no.		Positive rate by RT-PCR
				ORF3	S1	
1	HLJ2015/DP1-1	Apr-2015	Zhaodong/Heilongjiang/NECA	KU641637	KR351293	5/5
2	SD/QD/2015	Jun-2015	Qingdao/Shandong/ECA	KU641638	KU710223	3/3
3	BJ/2015/111	Nov-2015	Beijing/NCA	KU641639	KU710224	4/4
4	LN/SY/2015	May-2015	Shenyang/Liaoning/NECA	KU641640	—	1/1
5	HeB/CC/2015	Jul-2015	Chicheng/Hebei/NCA	KU641641	—	1/1
6	GD/MM/2015	Aug-2015	Maoming/Guangdong/SCA	KU641642	KU710225	7/9
7	HLJ/QQHR/2015	Sept-2015	Qiqihar/Heilongjiang/NECA	KU641643	KU710226	3/4
8	SC/CD/2015	Sept-2015	Chengdu/Sichuan/SWCA	KU641644	KU710227	6/7
9	SH/SG/2015	Sept-2015	Shanghai/ECA	KU641645	KU710228	4/4
10	HeB/2015/516	May-2015	Xinglong/Hebei/NCA	KU641646	KU710229	1/1
11	HeN/MY/2015	Sept-2015	Nanyang/Henan/CCA	KU641647	KU710230	5/5
12	SH/2015/122	Dec-2015	Shanghai/ECA	KU641648	—	1/2
13	HLJ/2015/116	Jan-2015	Daqing/Heilongjiang/NECA	KU641649	KU710231	3/3
14	TJ/2015/525	May-2015	Tianjin/NCA	KU641650	—	3/4
15	SD/LC/2015	May-2014	Liaocheng/Shandong/ECA	KU641651	KU710232	2/2
16	HLJ/2015/1228	Dec-2015	Hegang/Heilongjiang/NECA	KU641652	KU710233	3/3
17	SD/2015/415	Apr-2015	Liaocheng/Shandong/ECA	KU641653	KU710234	3/3
18	SD/YT/2015	Dec-2015	Yantai/Shandong/ECA	KU641654	—	4/10
19	HuB/YC/2015	Dec-2015	Yichang/Hubei/CCA	KU641655	KU710235	1/3
20	LN/DL/2015	Dec-2015	Dalian/Liaoning/NECA	KU641656	KU710236	3/3
21	AH/HF/2015	Dec-2015	Hefei/Anhui/ECA	KU641657	KU710237	1/1
22	JX/2015/1221	Dec-2015	—/Jiangxi/ECA	KU641658	KU710238	6/6
23	HuN/2015/1210	Dec-2015	—/Hunan/CCA	KU641659	—	1/1
24	HLJ/852/2015	Dec-2015	Baoqing/Heilongjiang/NECA	KU641660	KU710239	4/4
25	HeB/HS/2015	Dec-2015	Hengshui/Hebei/NCA	KU641661	—	1/2
26	SX/LL/2015	Dec-2015	Lvliang/Shanxi/NCA	KU641662	KU710240	6/6
27	HLJ/2015/1231	Dec-2015	Luobei/Heilongjiang/NECA	KU641663	KU710241	8/9
28	ShX/YA/2015	Dec-2015	Yanan/ShanXi/NWCA	KU641664	—	1/1
29	JX/2015/1224	Dec-2015	—/Jiangxi/ECA	KU641665	KU710242	9/9
30	HeB/TS/2015	Dec-2015	Tangshan/Hebei/NCA	KU641666	KU710243	7/12
31	SX/2015/121	Dec-2015	—/Shanxi/NCA	KU641667	—	2/2
32	HeB/2015/121	Dec-2015	Hengshui/Hebei/NCA	KU641668	—	2/2
33	FJ/FZ/2015	Dec-2015	Fuzhou/Fujian/SCA	KU641669	—	3/4
34	AH/XZ/2015	Dec-2015	Xuzhou/Anhui/ECA	KU641670	KU710244	7/10
35	HLJ/GQ/2015	Dec-2015	Hegang/Heilongjiang/NECA	KU641671	—	2/3
36	SH/2015/124	Dec-2015	Shanghai/ECA	KU641672	KU710245	2/3
37	HLJ/2015/1230	Dec-2015	Luobei/Heilongjiang/NECA	KU641673	KU710246	3/4
38	LN/TA/2015	Dec-2015	Taian/Liaoning/NECA	KU641674	KU710247	3/3
39	JL/2015/720	Jul-2015	Jilin/Jilin/NECA	KU641675	KU710248	1/1
40	SH/2015/921	Sept-2015	Shanghai/ECA	KU641676	KU710249	4/4
41	BJ/2015/516	May-2015	Beijing/NCA	KU641677	KU710250	1/1

*Note. NECA = Northeast China, ECA = East China, NCA = North China, SCA = South China, SWCA = Southwest China, CCA = Central China, and NWCA = Northwest China. The geographic coordinates of the collected samples (Farm, City or Province) are shown in the supporting information (S2 Table).

**Table 2:** Sequences analysis of *S1* and *ORF3* genes of PEDV strains identified in our study.

Selected strains (Genbank accession no.)		*ORF3*	*S1*
Identity of the PEDV strains identified in our study	nt	98.2%–100%	96.6%–100%
	aa	97.8%–99.6%	95.2%–99.5%
Compared with PEDV CV777 strain (AF353511)	nt	96.4%–97.2%	92.0%–92.7%
	aa	94.7%–96%	89.5%–91.3%
Compared with early PEDV S-INDEL strain LZC from China in 2006 (EF185992)	nt	95.1%–95.9%	91.4%–92.2%
	aa	93.3%–94.2%	89.2%–90.5%
Compared with variant virulent PEDV strain YN15 (passage 15 in Vero cells) from China in 2013 (KT021228)	nt	94.9%–96.1%	97.1%–98.5%
	aa	90.3%–91.7%	96.6%–98.5%
Compared with variant PEDV strain FL2013 (field) with reduced virulence from China in 2013 (KP765609)	nt	95.6%–96.1%	97.2%–98.5%
	aa	94.7%–96.0%	96.6%–98.6%
Compared with highly virulent PEDV strain CHYJ130330 from China in 2013 (KJ020932)	nt	98.5%–99.7%	96.8%–98.1%
	aa	98.7%–99.1%	95.7%–98.1%
Compared with recombinant PEDV strain CH/HNQX-3/14 from China in 2015 (KR095279)	nt	98.0%–99.2%	95.4%–96.9%
	aa	96.8%–97.7%	93.3%–95.5%
Compared with PEDV prototype strain USA/Colorado/2013 (high pathogenic strain) from USA in 2013 (KF272920)	nt	98.7%–99.9%	98.1%–99.5%
	aa	97.8%–99.1%	97.2%–99.7%
Compared with PEDV S-INDEL strain OH851 (low pathogenic strain) from USA in 2014 (KJ399978)	nt	98.7%–100%	93.0%–94.7%
	aa	98.2%–99.6%	91.3%–93.9%

**Fig 1:**
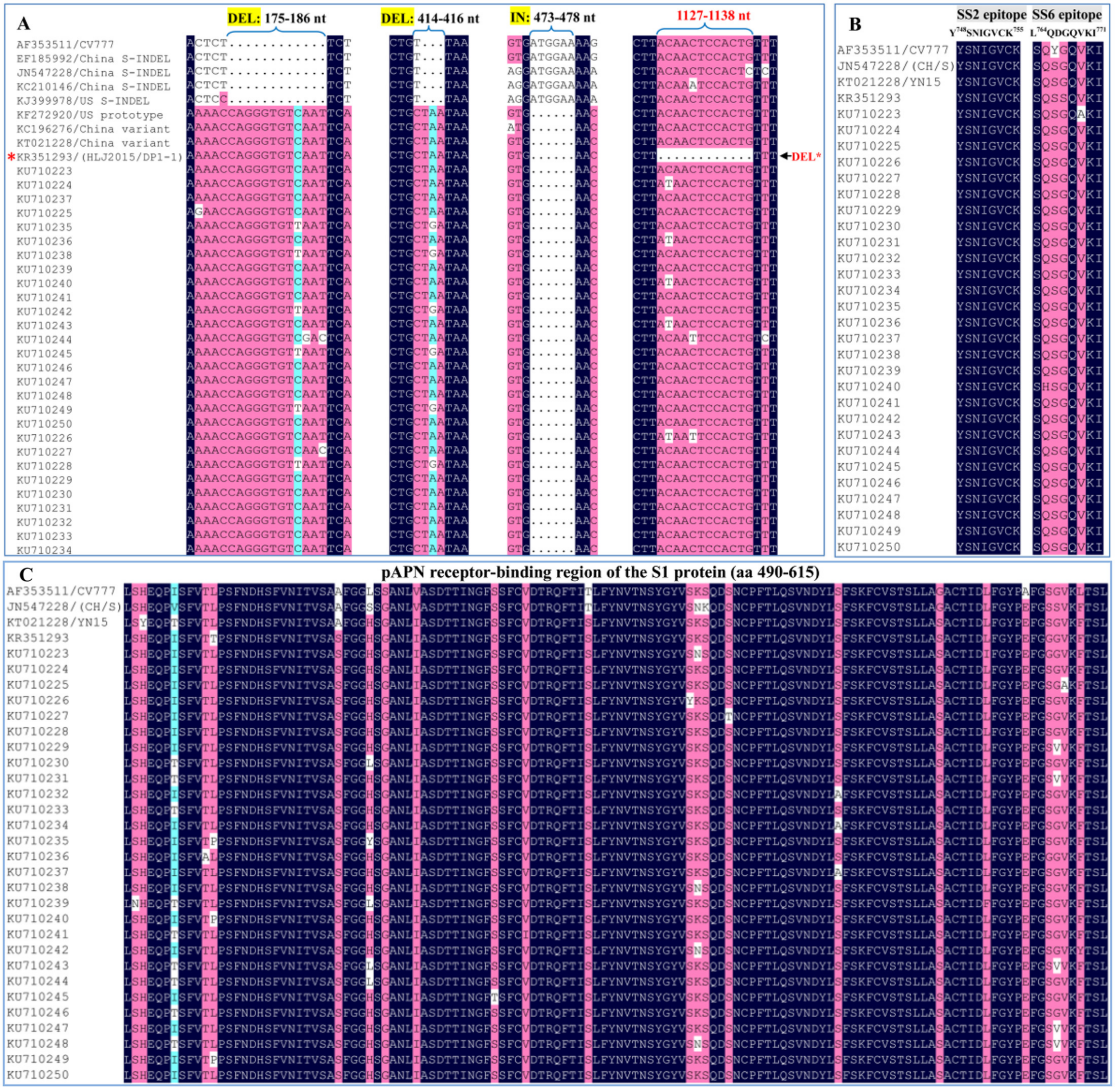
Analysis of the type S-INDEL strain, epitopes, and pAPN receptor-binding region of the 29 *S1* genes identified in our study. (A) Analysis of the type S-INDEL strain in the identified 29 *S1* genes of PEDV. (B) Analysis of the epitopes SS2 (Y^748^SNIGVCK^755^) and SS6 (L^764^QDGQVKI^771^) in the identified 29 *S1* genes of PEDV. (C) Analysis of pAPN receptor-binding region (aa 490–615) in the identified 29 *S1* genes of PEDV.

To further reveal the molecular characterization of the 29 identified *S1* genes, the type S-INDEL strains, epitopes, and the pAPN receptor-binding regions were analyzed (Fig 1). The 29 identified PEDV strains were classified as the global non S-INDEL strains. The *S1* gene of the HLJ2015/DP1-1 strain has a unique deletion of 12 nucleotides at position of 1130–1141 (using CV777 as the reference strain) when compared with S-INDEL and non S-INDEL strains (Fig 1A). The SS2 epitope (Y^748^SNIGVCK^755^) was found to be highly conserved in the 29 identified *S1* genes, while two amino acid substitution occurred in the SS6 epitope (S^764^QSGQVKI^771^) when compared with the CV777 strain (Fig 1B). A total of 7 amino acid (aa) substitutions occurred in pAPN receptor-binding region (aa 490–615) of all identified PEDV *S1* genes when compared to the CV777 strain (Fig 1C).

### Phylogenetic analysis of PEDV

**Fig 2:**
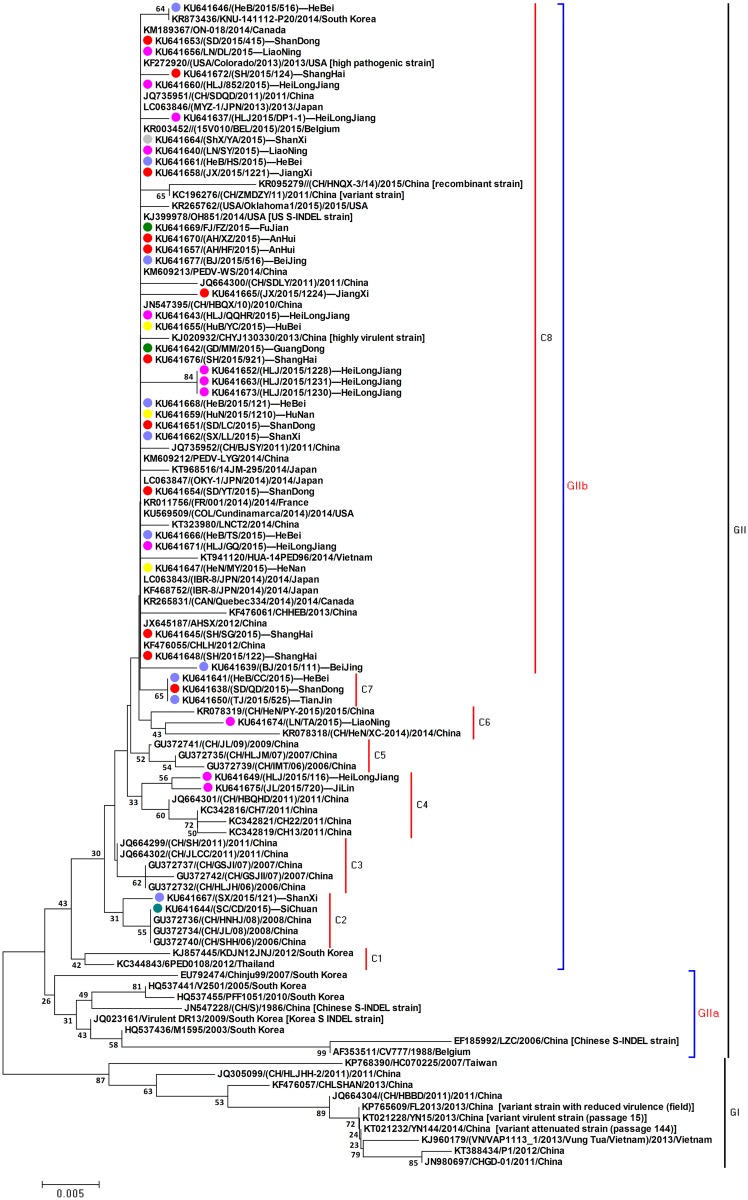
Phylogenetic analysis of the identified PEDV strains on the basis of *ORF3* gene sequences. *Note*. The classical PEDV strain CV777, North American (NA) prototype strain USA/Colorado/2013 (non S-INDEL), US S-INDEL strain OH851, Chinese early PEDV strains (CH/S), Chinese PEDV field strains (2006–2015), and other countries PEDV field strains were choose for genotyping and phylogenetic analysis of the 41 *ORF3* genes identified in our study. Light purple dot represents PEDV strains identified in North China; red dot represents PEDV strains identified in East China; pink dot represents PEDV strains identified in Northeast China; grey dot represents PEDV strains identified in Northwest China; green dot represents PEDV strains identified in South China; yellow dot represents PEDV strains identified in Central China; blue green dot represents PEDV strains identified in Southwest China.

Phylogenetic analysis using the nucleotide sequences of the *ORF3* gene revealed that the 41 identified PEDV strains had a close relationship with Chinese field strains (identified between 2011 and 2015), several North American strains, other Asian strains, and several European strains, and differed from the CV777 strain as well as early PEDV strains from China, South Korea and Japan (Fig 2). The 41 PEDV strains identified in our study formed eight clusters in the *ORF3* gene–based phylogenetic tree, in which the PEDV strains collected from same region of China showed genetic diversity from each other. Phylogenetic analysis of *ORF3* did not reveal any differences between the S-INDEL and non S-INDEL PEDV strains.

**Fig 3:**
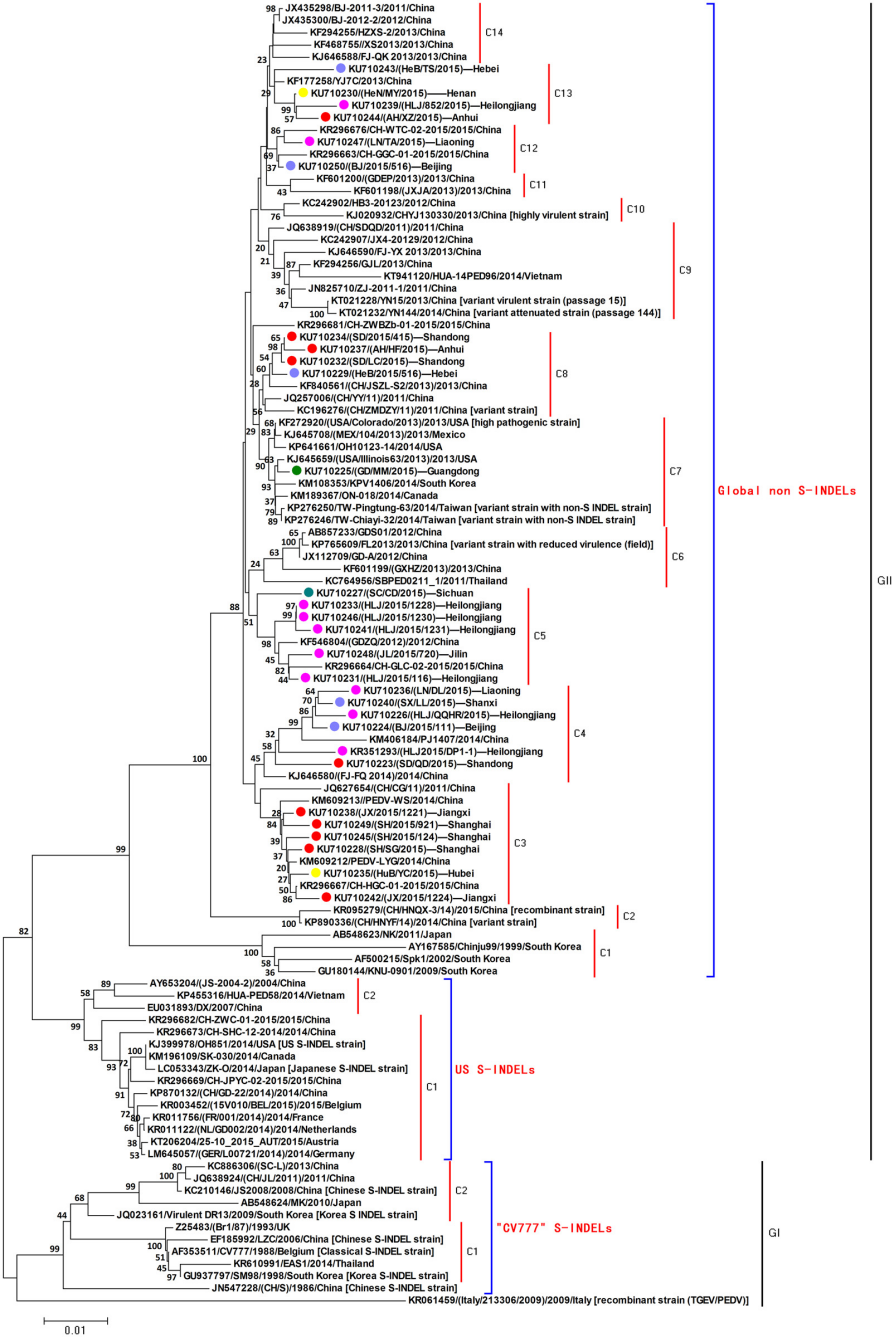
Phylogenetic analysis of the identified PEDV strains on the basis of *S1* gene sequences. *Note*. The “CV777” S-INDEL strain, North American (NA) prototype strain USA/Colorado/2013 (non S-INDEL), US S-INDEL strain OH851, Chinese S-INDEL strains (LZC, JS2008, and CH/S), Japanese S-INDEL strain ZK-O, South Korea S-INDEL strains (SM98 and virulent DR13), European S-INDEL strains (FR/001/2014, 15V010/BEL/2015, and GER/L00721/2014), Chinese PEDV field strains, and other countries non S-INDEL strains were choose for genotyping and phylogenetic analysis of the 29 *S1* genes identified in our study. Light purple dot represents PEDV strains identified in North China; red dot represents PEDV strains identified in East China; pink dot represents PEDV strains identified in Northeast China; grey dot represents PEDV strains identified in Northwest China; green dot represents PEDV strains identified in South China; yellow dot represents PEDV strains identified in Central China; blue green dot represents PEDV strains identified in Southwest China.

The phylogenetic tree drawn from the *S1* gene sequences was comprised of two groups (GI and GII). The GI group included the “CV777” S-INDEL PEDV strains from China, South Korea, and the prototypical CV777 strain. While the GII group was split into two subgroups, with the “US” S-INDEL strains and the global non S-INDEL strains clustering apart. The US S-INDEL subgroup included the Japanese S-INDEL strain, the European S-INDEL strains, and several field strains from China, Canada and Vietnam. The 29 strains identified in our study as well as reference strains from China, North America, and other Asian countries fell into the non-INDEL subgroup (Fig 3). This analysis revealed that the 29 identifed PEDV strains were closely related to known Chinese variant strains and the highly pathogenic USA prototype strain USA/Colorado/2013, and formed fourteen different clusters.

### Analysis of recombination and variation of PEDV

**Fig 4:**
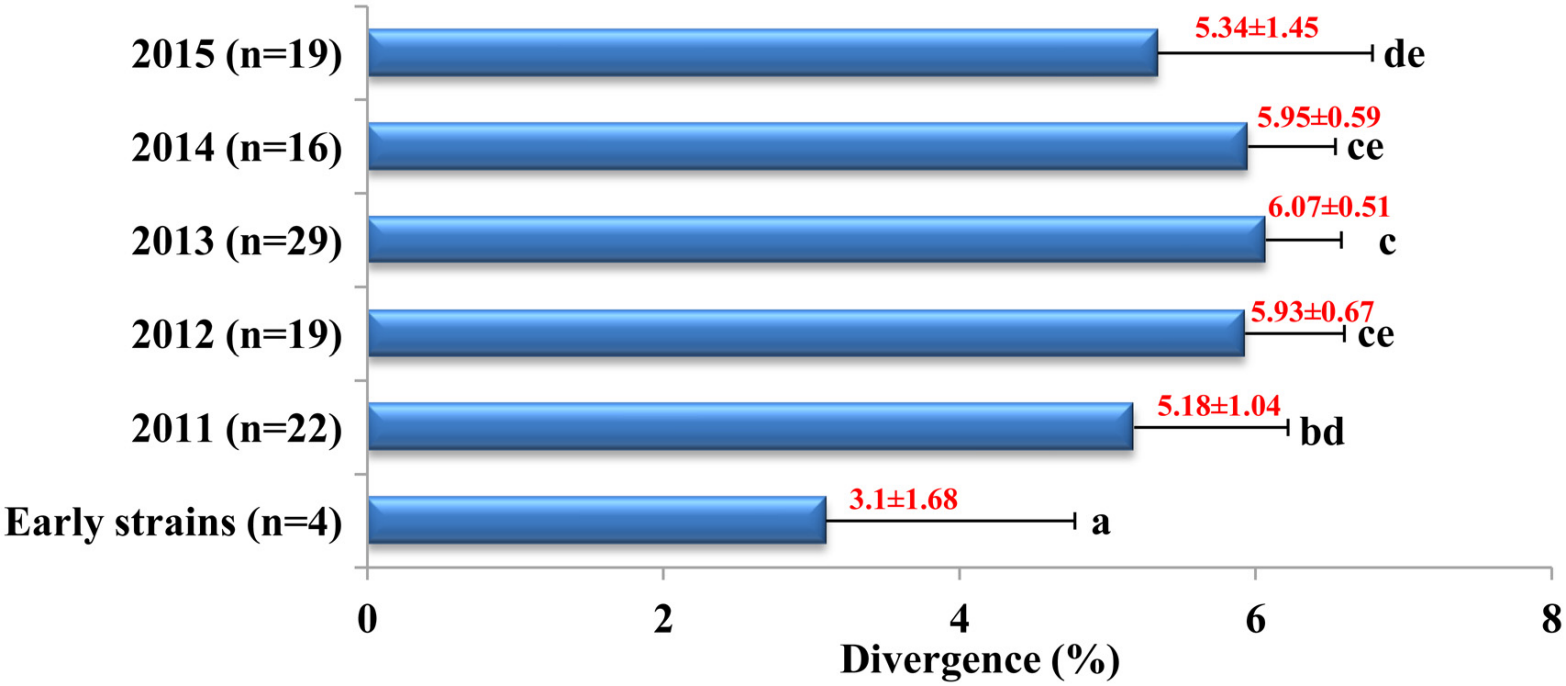
Divergence analysis of complete *S* gene of the selected Chinese PEDV strains. *Note*. The values (red) represent “average value ± standard deviation” of the nucleotide divergence (%) of each group; the different letters represent statistically significantly difference (*P*<0.05) among six groups, and the same letters (one or more) represent no significant difference (*P*>0.05) among six groups.

The recombination analysis of the *S1* gene revealed that nine potential recombination events occurred between the 29 strains identified in this study and three early Chinese PEDV strains. The breakpoint positions for the recombination events are shown in the supporting information (S1 Table). Of the nine potential recombination events, six recombination events occurred among the 29 identified PEDV strains, and three recombination events occurred between these 29 PEDV strains and three early Chinese reference strains (Chinese S-INDEL strains) (Table 3). To explore the genetic variation of PEDV strains, the divergence (%) within the *S* gene was analyzed. The *S* gene of the prototype CV777 strain was used as the comparator. This data is shown in the supporting information (S3 Fig). Divergence of 5.18%–6.07% within the selected Chinese PEDV field strains (2011–2015) was significantly (*P<*0.05) higher than the divergence (3.1%) of the early Chinese PEDV strains. There was a trend for increased divergence in the selected Chinese PEDV field strains from 2011 to 2013 but there was a trend toward decreased divergence in the selected Chinese PEDV field strains from 2013 to 2015 (Fig 4).

**Table 3:** Potential recombination analysis between the 29 identified *S1* genes and the 3 *S1* genes from early Chinese PEDV strains.

Potential recombinant events	Recombinant sequences (group^※^)	Major parent sequences (group^※^)	Minor parent sequences (group^※^)	Breakpoint Positions in recombinant sequences	Program or method of recombination detection
1	KU710237 (non S-INDEL)	KU710230 (non S-INDEL)	KU710249 (non S-INDEL)	1039–2374	Maxchi (*P* = 3.69E-04); Chimaera (*P* = 1.65E-04); SiSscan (*P* = 1.35E-04); 3Seq (*P* = 4.07E-06).
2	KU710249 (non S-INDEL)	KU710223 (non S-INDEL)	KU710244 (non S-INDEL)	355–976	Maxchi (*P* = 1.36E-04); Chimaera (*P* = 1.04E-03); 3Seq (*P* = 2.25E-03).
3	KU710244 (non S-INDEL)	KU710227 (non S-INDEL)	KU710230 (non S-INDEL)	315–2356	Chimaera (*P* = 3.91E-11); 3Seq (*P* = 2.03E-03).
4	KU710225 (non S-INDEL)	KU710236 (non S-INDEL)	KU710230 (non S-INDEL)	1207–2336	Maxchi (*P* = 3.08E-02); Chimaera (*P* = 3.22E-03); SiSscan (*P* = 5.33E-07).
5	KU710223 (non S-INDEL)	KU710240 (non S-INDEL)	KU710248 (non S-INDEL)	589–1747	Maxchi (*P* = 1.21E-02); SiSscan (*P* = 4.63E-04).
6	KR351293 (non S-INDEL)	KU710248 (non S-INDEL)	KU710240 (non S-INDEL)	705–1759	Maxchi (*P* = 4.57E-02); SiSscan (*P* = 1.60E-05).
7	EF185992 (S-INDEL)	KU710247 (non S-INDEL)	JN547228 (S-INDEL)	46–808	GENECONV (*P* = 6.19E-04); Maxchi (*P* = 8.65E-07); Chimaera (*P* = 2.5E-02); SiSscan (*P* = 1.22E-25).
8	KC210146 (S-INDEL)	KU710233 (non S-INDEL)	JN547228 (S-INDEL)	62–665	Maxchi (*P* = 2.49E-05); Chimaera (*P* = 4.83E-03); SiSscan (*P* = 1.48E-22).
9	KU710230 (non S-INDEL)	JN547228 (S-INDEL)	KU710248 (non S-INDEL)	23–631	Maxchi (*P* = 5.59E-04); SiSscan (*P* = 5.13E-31).

^**※**^Group was defined based on the phylogenetic analysis of *S1* gene of the selected PEDV strains in our study (see Fig 3).

## Discussion

In this study, we collected 165 samples from 41 PEDV-positive farms in seven regions of China during 2015. After sequencing of the *ORF3* and *S1* genes, the molecular characterization of the identified PEDV strains was analyzed. Sequence analysis of both the *ORF3* and *S1* genes revealed that the PEDV strains identified in our study exhibited high similarity to the Chinese PEDV variant reference strains when compared with the CV777 prototype strain or early Chinese PEDV strains. Phylogenetic analysis showed that these identified PEDV strains have different genetic lineages, but that all of the strains have a close relationship with other non-INDEL strains, and are distinct from the “CV777” and “US” S-INDEL strains. Previous reports showed that the PEDV S-INDEL strain had a lower pathogenicity than the North American prototype strain [13, 14]. In our study, the identified PEDV strains and selected Chinese PEDV reference strains were divided into three strain types: (1) “CV777” S-INDELs (early Chinese PEDV strains), (2) “US” S-INDEL (Chinese PEDV field strains), and (3) non S-INDEL (Chinese PEDV field strains) strains. These groupings were similar to those observed for Japanese PEDV strains. This result demonstrates that the PEDV strains from China and Japan are more genetically diverse than those from North American countries. In our study, these PEDV reference strains belonging to the “US” S-INDEL subgroup showed a close relationship to the PEDV strains that fall within the global non S-INDEL subgroup, and differed genetically from the PEDV strains belonging to the “CV777” S-INDEL subgroup. The differences in the pathogenicity among the “CV777” S-INDEL subgroup, the “US” S-INDEL subgroup, and the global non S-INDEL subgroup are an interesting topic and require further studies. Additionally, co-circulation of S-INDEL and non S-INDEL strains should be investigated in an effort to better understand the genetic and pathogenic variability of global PEDV strains.

The PEDV strain USA/Colorado/2013 is a North American (NA) prototype strain with high pathogenicity; while the OH851 strain, an S-INDEL strain, is much less pathogenic [12, 13]. All the PEDV strains identified in our study were genetically distinct from the “US” S-INDEL subgroup which includes OH851, and instead clustered with the highly virulent non S-INDELs subgroup which contains the USA/Colorado/2013 strain. This clustering suggests that those PEDV strains identified in our study should possess high pathogenic potential. However, Chinese highly virulent strains (CHYJ130330 and YN15), Chinese variant attenuated strain (YN144), and Chinese variant strain with reduced virulence (FL2013) were also found in the same subgroup [24–26]. This data implies that the pathogenicity of these Chinese PEDV strains and the type strain of the non S-INDEL subgroup can diverge in pathogenic potential, which means that phylogenetic clustering is not sufficient on its own to predict virus pathogenicity, which would make this an ideal topic for future research. Meanwhile, sequence analysis of *ORF3* and *S1* genes may not be sufficient to fully reveal the genotype of pathogenic PEDV strains.

Antigenic epitopes and receptor-binding domain represent two important biological functions of the PEDV S protein [20, 27]. In our study, the SS2 epitope (Y^748^SNIGVCK755) was found to be highly conserved in the 29 identified PEDV strains, which is in line with previous studies [7, 28, 29]; while the SS6 epitope (S^764^QSGQVKI771) exhibited amino acids substitutions, all of which have been reported in other studies [7, 28, 29]. The C-terminal domain (CTD) of the S1 domain is bound to porcine aminopeptidase N (pAPN) [27], and when the pAPN receptor-binding region of the 29 identified *S1* genes was evaluated, we identified 7 different amino acid substitutions when compared with the PEDV CV777 strain. Amino acids substitutions within the SS6 epitope and pAPN receptor-binding region are presumed to be responsible for pathogenic changes of PEDV [21, 30]. Recently, the PEDV variant strains with large deletions in the *S* gene have been reported in South Korea and Japan [31, 32]. Similar mutant strains have not yet been reported in the pig population of China. In our study, the HLJ2015/DP1-1 strain contains a novel twelve nucleotide deletion (A^1130^CAACTCCACTG1141) within the *S1* gene when compared with the CV777 strain, other non S-INDEL strains, and S-INDEL strains. The 12 nucleotide deletion within the *S1* gene was further confirmed as unique when a global survey of PEDV strains was undertaken using the nucleotide BLAST tool from the NCBI. The *S1* gene of the strain HLJ2015/DP1-1 was identified in a vaccinated population suffering from long-term diarrhea caused by a single PEDV infection (data not shown). This unique deletion of the *S* gene was relatively rare in the global non S-INDEL strains, and may result from long-term immune pressure.

Since the outbreak of SARS-CoV and MERS-CoV, the recombination and variation of the coronavirus, especially as it concerns the cross species transmission of the coronavirus, has become a hot research topic [33]. In all of these coronaviruses, the S protein initiates infection by mediating receptor-recognition and membrane fusion and plays a key role in tracing the evolution of coronaviruses. In the past, phylogenetic analysis and identification of nucleotide or amino acid substitutions within the *S* gene were widely used for evaluating the genetic evolution of PEDV. There were little quantitative measures for evaluating PEDV variation. In our study, the divergences were identified by comparison of specific target sequences within the *S* gene between the selected Chinese PEDV field strains and the prototype CV777 strain. Using this approach, we were able to show that the selected Chinese PEDV field strains between 2011 and 2015 show a significant increase in diversity when compared to early Chinese PEDV strains, which is in line with previous reports about PEDV variants [4]. The amount of genetic divergence within the selected Chinese PEDV strains showed a downward trend from 2013 to 2015, which may be associated with recent effective control strategies for PEDV in China. Recombination plays a pivotal role in the evolution of coronaviruses, it allows the emergence of new strains with altered virulence, and potentially broader host ranges [9]. In our study, six out of a possible nine recombination events occurred between the 29 identified non S-INDEL strains. The remaining three recombination events occurred between the 29 identified non S-INDEL strains and three early Chinese S-INDEL strains. Recently, the recombination between PEDV and TGEV has generated a new swine enteric coronavirus which has been reported in pig population in Italy [9], confirming the potential for increased or altered virulence in PEDV as a result of recombination. The limited data presented here demonstrates that there is a potential for recombination and that this can occur between both related and unrelated coronaviruses.

## Conclusions

This study has identified 41 PEDV strains in seven regions of China in 2015. These strains belong to the non S-INDEL strains, and exhibit genetic diversity as well as evidence of potential recombination. Amino acid substitutions have occurred in both the SS6 epitope and pAPN receptor-binding region; and one PEDV strain was shown to harbor a unique deletion (A^1130^CAACTCCACTG1141) within the *S1* gene. Phylogenetic analysis revealed that three types PEDV strains, non S-INDEL, “CV777” S-INDEL and “US” S-INDEL, co-circulated in swine population in China. However, further studies are needed to clarify the effect of the phylogenetic grouping on the virulence of these PEDV strains.

## Supporting Information

S1 Fig Nucleotide sequences of the PEDV *ORF3* gene for generation of phylogenetic tree. (TXT)

S2 Fig Nucleotide sequences of the PEDV *S1* gene for generation of phylogenetic tree. (TXT)

S3 Fig The data of the divergence analysis of *S* gene from Chinese PEDV strains. (XLS)

S1 Table The breakpoint positions of potential recombination events in our study. (XLS)

S2 Table The geographic coordinates of the collected samples (Farm, City or Province). (XLS)
